# Role of VATS-US in identifying and characterizing pulmonary nodules: a narrative review

**DOI:** 10.3389/fsurg.2025.1567390

**Published:** 2025-05-20

**Authors:** Susana González-Suárez, María Grao Roca, Juan-Camilo Vivas, Alberto Jauregui

**Affiliations:** ^1^ Department of Surgery, Universitat Autònoma de Barcelona, Barcelona, Spain; ^2^ Department of Anesthesia and Intensive Care, University Hospital Vall d'Hebron, Barcelona, Spain; ^3^ Cardiovascular Diseases Research Group, Vall d'Hebron Institut de Recerca (VHIR), Barcelona, Spain; ^4^ Department of Anesthesia and Intensive Care, University Hospital Vall d´Hebron, Barcelona, Spain; ^5^ Department of Thoracic Surgery and Lung Transplant, University Hospital Vall d´Hebron, Barcelona, Spain; ^6^ Department of Surgery, Universitat Autònoma de Barcelona, Barcelona, Spain

**Keywords:** VATS-US, pulmonary nodules, echogenic pattern, nodule identification, nodule characterization

## Abstract

The aim of this study was to show the efficacy described in the scientific literature of lung ultrasound (LU) during video-assisted thoracic surgery (VATS) to determine the location and characterization of pulmonary nodules. The results showed that intraoperative LU is especially useful in localizing lung lesions by VATS with a sensitivity close to 100%. It was also shown to be useful in planning very precise lung resections, reducing the resection of healthy lung tissue. Although general criteria could be established for the degree of benignity/malignancy of lung lesions based on the ultrasound patterns obtained, the great variability observed in these patterns does not offer sufficient guarantees to make a reliable diagnosis. In this sense, the application of Doppler ultrasound or the utilization of Intraoperative Contrast-Enhanced Ultrasound (Io-CEUS) in a completely collapsed lung can be advantageous for the accurate localization and characterization of pulmonary lesions. Even so, at present, definitive confirmation of the nature of lung lesions usually requires biopsy and histopathological study. The development of artificial intelligence algorithms that integrate the results of histological analyses with various types of ultrasonographic patterns (based on pulmonary aeration obtained, as well as Doppler and Io-CEUS records) will likely represent the future of ultrasonographic differentiation of these lesions.

## Introduction

Since its introduction in 1990, video-assisted thoracic surgery (VATS) has become increasingly prevalent. This minimally invasive technique involves accessing the thoracic cavity through an incision of less than 4 cm, enabling visualization and endoscopic instrumentation of the lung without the need to sever muscles, as is necessary in traditional thoracotomy. Due to its less invasive nature, VATS offers several advantages, including reduced morbidity (1), shorter hospital stays (1), diminished postoperative pain, and improved oncologic outcomes (2), particularly in patients with limited respiratory reserve. Despite these benefits, VATS requires specialized training (3) due to the constrained surgical access and challenges in locating pulmonary lesions, especially when dealing with underlying pulmonary conditions, small lesions, or those situated in deep, basomedial, or postero-medial regions (4, 5).

In this context, lung ultrasonography has proven to be a valuable tool (6, 7). The use of ultrasound is expanding across various medical fields, including cardiology (8), internal medicine (9), anesthesiology, and intensive care (10). It is also instrumental in examining pleural and subpleural lesions, guiding the placement of thoracic drains (11, 12), and performing percutaneous needle biopsies of the lung (13, 14). In the postoperative setting, ultrasound has reduced the reliance on x-rays (15) by effectively detecting atelectasis, pleural effusion, pneumonia, pneumothorax, and diaphragmatic dysfunction with sensitivities of 88%, 100%, 88%, 100%, and 91%, respectively, and specificities of 92%, 100%, 86%, 82%, and 91% (16–20).

During VATS, ultrasonography (VATS-US) has been introduced to locate and differentiate pulmonary nodules and their adjacent structures (21, 22). Since it can be performed directly on the lung surface through the thoracoscopic ports, it provides a direct and detailed visualization of the lung parenchyma, as the probe is in direct contact with the lung and free from the interference of air in the lung tissue, provided a complete lung collapse is achievable (23). Moreover, no complications have been reported in the use of intraoperative LU for identifying pulmonary nodules. However, other techniques, such as the insertion of a hooked wire or metal coils, technetium-99-labeled human serum albumin microspheres, and radiotracer-guided thoracoscopic biopsy, while achieving success rates of up to 100% and reducing the need for open thoracotomy in more than 50% of cases, are associated with notable complications including air embolism, pneumothorax, and intraparenchymal hemorrhage (24–30). In addition, technical difficulties may arise during the execution of these pre-surgical tests. Issues such as failed hook wire placement, migration of the coil between its insertion and the time of surgical resection (occurring in 3%–10% of cases), or the injection of methylene blue far from the lung lesion, can result in unsuccessful nodule localization if displacements exceed 10 mm (28, 31–33).

To evaluate the efficacy of VATS-US in the localization and characterization of pulmonary nodules, and to identify potential measures for improving nodule identification rates, a comprehensive literature search was conducted using the Medical Literature Analysis and Retrieval System Online (MEDLINE-PubMed), EMBASE (Elsevier), and the Web of Science Core Collection (Clarivate). This review assessed variables including the ultrasonographic patterns of pulmonary nodules, the size and depth of the nodules, the types of ultrasonographic probes used, the results of Doppler and Intraoperative Contrast-Enhanced Ultrasound (Io-CEUS recordings, the correlation between ultrasonographic findings and lesions identified via CT and/or histological analysis, as well as the limitations of the ultrasonographic technique.

## Efficacy of VATS-US for the localization of pulmonary nodules

### Comparison of VATS-US with traditional methods of palpation and visual inspection for pulmonary nodule localization

Most solid pulmonary nodules can be palpated and/or visualized. Manual palpation of lung lesions is the oldest method used and can achieve a 100% success rate in detecting solid lung metastases during conventional thoracotomies, although it carries a false-positive rate of 13–48.5% (34). It can even detect lung metastases that were not identified by preoperative CT scans (35). However, manual palpation presents challenges during VATS and is rarely used as the sole technique for localizing pulmonary nodules. Instead, it is typically combined with other techniques, such as intraoperative ultrasound. According to the literature, the percentage of lung lesions detected by ultrasound during VATS ranges from 76% to 100% (20, 36, 37). Some authors have reported detecting more nodules using VATS-US than with VATS alone or simple manual palpation (37, 38). Kondo et al. (23) also achieved a higher sensitivity with lung ultrasonography (93%) compared to palpation (40%). Khereba et al. (39) found that, during VATS, 93.5% of pulmonary nodules were detected by ultrasound, compared to 27% detected by visual inspection, 40% identified by digital palpation, and 38% by palpation with instrumental slide techniques. The sensitivity of VATS-US was 93%, with a positive predictive value of 100%. In 20 cases (43%), pulmonary nodules were not identified by any of the traditional methods and were only detected by VATS-US, thus avoiding conversion to thoracotomy or lobectomy (39). Hou et al. (40) demonstrated successful localization of pulmonary nodules in 97% of patients who underwent intrapulmonary ultrasonography, compared to only 48.5% in those who underwent lung palpation (*p* < 0.05). This success rate with intrathoracic ultrasonography is comparable to that reported by other authors using alternative methods for nodule identification, such as CT-guided metal hook insertion, methylene blue or isotope injection, and intraoperative tracheal endoscopic magnetic navigation (40–43).

Overall, it should be noted that although VATS-US adds an additional 10–15 minutes to the surgery, its use could reduce the need for conversion to thoracotomy by up to 59% of cases (44–48). Furthermore, compared to the manual palpation method, intraoperative ultrasonography demonstrated an even shorter time for lesion identification, with an average of 7.09 ± 1.80 minutes for ultrasonography vs. 9.67 ± 2.62 minutes for palpation (*P* < 0.05), thus improving the feasibility of minimally invasive surgery (39). Some authors, such as Hou et al. (40), considered the palpation method to have failed if it took more than 12 minutes to identify the lesions. The rationale for this decision was based on various factors, beyond simply avoiding prolonged surgical time. In their experience, the likelihood of detecting lung lesions decreased as palpation time increased, and there is scientific evidence indicating that excessive surgical duration elevates the risk of tumor metastasis (49, 50).

### Effectiveness of pulmonary ultrasound depending on the size and depth of pulmonary nodules

Thanks to VATS-US, some authors have successfully identified very small pulmonary nodules, even those smaller than 2 mm, as well as deeply located nodules (39, 45). Khereba et al. (39) identified peripheral nodules (not in contact with the visceral pleural line on CT) as small as 2 mm. Other authors, such as Piolanti et al. (7), although able to detect two small nodules that had not been identified by helical CT, were unable to identify a very peripheral subpleural lesion measuring 3 mm in diameter. Therefore, when a lesion is located very peripherally, its detection with ultrasound may be missed. In such cases, the use of high-frequency ultrasound transducers could prove helpful, in addition to visual inspection of the lung surface by the surgeon. However, in general, most peripheral pulmonary nodules can be identified.

Other authors have also been able to identify small nodules located intraparenchymally within the lung. In ex vivo samples, Fiorelli et al. (51) detected nodules up to 7 mm in diameter at a depth of 5 mm. Using VATS-US, Gambardella et al. (52) identified deep subcentimeter intraparenchymal nodules (less than 1 cm in diameter, with a mean size of 7.3 ± 1.2 mm) located at a distance of 16.2 ± 2.4 mm from the visceral pleura. Taurchini et al. (46) also demonstrated a high sensitivity (100%) of LU in detecting small pulmonary nodules less than 2 cm in size and centrally located (53). Furthermore, LU was even able to identify small nodules that had not been previously detected by preoperative CT (7, 37, 47, 48) (Table 1).

**Table 1:** Location and characterization of pulmonary nodules according to various authors.

Authors. Number of patients	Intraoperative data of VATS-US. Nodule size	Characterization of nodules. Comments
Bastone et al. (80) - 2024, Italy. Retrospective study - *N* = 31 patients with solid lung nodule < 2 cm and a distance between the nodule and the pleural surface ≥ 2 cm - Patients excluded: patients with pulmonary nodules ≥2 cm and a distance between visceral pleura and nodule < 2 cm, patients with a high grade of pulmonary emphysema	- 4–13 MHz probe with flexible tip - VATS-US: Performed by a certified operator Investigated 31 pulmonary nodules Identified 31 pulmonary lesions (Sensitivity 100%). Median nodule diameter 11 mm (9–13) Distance from pleural surface 27 mm (25–28)	- Solitary and deep-sited solid pulmonary nodules - Uniportal VATS-US was faster and more effective than conventional techniques (palpation) in multiportal VATS for nodule detection
Gambardella et al. (52) - 2023. Italy. Retrospective study - *N* = 43 patients with solitary and deep-seated pulmonary nodules < 1 cm - Excluded patients: Patients with recent myocardial infarction or unstable angina, impossibility to tolerate single lung ventilation, emphysema, nodules close to the hilar zone - CT detected 43 pulmonary nodules	- 4–12 MHz convex probe - VATS-US: Performed by thoracic surgeons who had performed over 300 thoracic oncology procedures using VATS and were experienced in pulmonary ultrasound Investigated 43 nodules Identified 43 nodules (Sensitivity 100%) Nodules diameter 7,3 ± 1,2 mm Distance from pleura surface16,2 ± 2,4 mm	Solitary and deep-seated pulmonary nodules <1 cm - Intracavitary VATS-US is an effective method of localization of parenchymal lung nodules during selected wedge resection procedures
Hou et al. (40) - 2020, China. Prospective study - *N* = 33 patients - Excluded patients: Patients with nodules close to the hilar zone and no obvious pleural traction signs found by thoracoscopy. - CT detected 33 pulmonary nodules (mean size 13,21 ± 3.37 mm (range 8–20) and distance from pleural line 14,64 × 5,58 mm	- 7.5–10 MHz linear probe - VATS-US Performed by senior physician specializing in lung ultrasound Investigated 33 nodules Identified 32 nodules (Sensitivity of 97%). One pure GGO nodule of 10 mm located 10 mm under the visceral pleura was not identified in a patient with emphysema. Nodules diameter 8,69+−2,60 mm (13,5–5) and had a good correlation with the diameter on CT	- Solids = 14. These nodules could easily be visualized by US showing a hypoechoic pattern with associated posterior acoustic enhancement - Pure GGOs = 10 - Mixed GGOs = 9 - VATS-US can locate pulmonary nodules efficiently and safely
Khereba et al. (39) - 2012, Canada. Prospective study - *N* = 43 patients with 46 nonsubpleural parenchymal pulmonary nodules (not touching the visceral pleura on the CT) - Excluded patient with pulmonary nodules believed to be easily localized during VATS (large size, pleural retraction) - CT identified 46 nodules	- 5–10 MHz linear probe with articulated head - VATS-US Performed by 4 thoracic surgeons, one knowledgeable and the other with no formal training or experience Investigated 46 nodules Identified 43 nodules (Sensitivity 93.5%). Undetectable pulmonary nodules: Hodgkin lymphoma nodule (lung parenchyma was edematous and thickened); another palpable nodule; and a third nodule near the spine. Mean nodule dimension 11.3 mm in the long axis (range 2.7–27.7) and 8.6 mm in the short axis (range 2–20.4) Distance from pleural surfce: 1–24.1 mm The site of the nodules found by VATS-US was correlated with the findings of CT scan.	- Non-subpleural nodules - In 20 cases, nodules were not identifiable with any of the standard VATS techniques and they were only found with VATS-US - VATS-US prevented conversion to thoracotomy or lobectomy in 43.5% (20/46) of cases - In one patient single lung ventilation was not possible owing to hypoxia. In this patient, VATS-US was able to localize the nodule without lung isolation
Kondo et al. (23) - 2009. Japan. Prospective study - *N* = 44 patients - Excluded patients: patients with chronic obstructive pulmonary disease or asthma, pulmonary fibrosis, severe heart disease - CT detected 53 nodules, (≤ 20 mm diameter) - A preoperative hook-wire technique was used to localize pure GGOs that were less than 10 mm in diameter and did not involve the visceral pleura (13 patients)	- 7.5 or 18 MHz linear probe - VATS-US: Investigated 53 nodules Identified 53 GGOs nodules (20 mixed and 33 pure) (Sensitivity 100%)	- GGOs nodules (20 mixed and 33 pure) - A strong correlation was found between the resection margins measured by ultrasonogram and the margins determined by histologic examination - Correlation between the echo types and a complete deflation of the lung (but not with size, histologic type, or presence of solid components) - In patients with complete collapse (30 cases), the GGO produced hyperechoic pattern - If a complete deflation could not be obtained, GGO showed high echo images (9 GGO) and low echo images (7 GGO). In these cases, intraoperative ultrasound can effectively localize GGOs - All instances of acoustic shadow were attributed to high internal echo images, whereas all the acoustic enhancements were ascribed to the presence of low internal echo images. - The presence of a posterior echo might be related to the size of the GGO (the larger the GGO, the larger the after-echo)
Lesser et al. (65) - 2010, Germany. Prospective study - *N* = 155 patients with close contact of the tumor [non-small cell lung cancer (NSCLC)] with mediastinal structures (T4, pulmonary artery, left or right atrium, superior vena cava, thoracic aorta) by CT and questionable resectability. - Excluded patients: patients with non-advanced lung cancer - CT detected 155 nodules	- 5, 7, 7,5 MHz convex probe - VATS-US: - . Investigated 155 nodules Identified 155 nodules (Sensitivity 100% for determining the location of nodules and 94.1% for determining operability) VATS-US did not detect operability in 3.9% (6 patients) of tumors affecting the left atrium	- Nodules = NSCLC - A strong correlation was found between the resection margins measured by ultrasonogram and the margins determined by histologic examination - Estimation of operability in locally advanced lung cancer can be improved with VATS-US compared with thoracoscopy alone and CT
Matsumoto et al. (37) - 2004, Japan. Prospective study - *N* = 23 patients - CT detected 25 nodules (10–30 mm)	- 7.5 MHz linear probe - VATS-US: Performed by thoracic surgeon and 2 radiologists Investigated 25 nodules Identified 25 nodules (Sensitivity 100%)	- Small peripheral pulmonary nodules - VATS-US could detect nodules ≤ 10 mm in diameter if they were located < 15 mm from the pleural surface - VATS-US may allow differential diagnosis between malignant and non-malignant - Malignant nodules: heterogenous pattern and an ill-defined margin (homogeneous pattern in minor proportion) - Benign: Predominantly homogeneous pattern with well-defined margin
Mattioli et al. (22) - 2015, Italy. Prospective study. - *N* = 54 patients. - No patient excluded - CT detected 65 nodules, median diameter 15 mm (range 3–35 mm).	- 7.5–10 MHz linear probe with flexible tip - VATS-US: Performed by surgeons (after an initial learning curve in collaboration with the radiology staff) Investigated 45 nodules Identified 42 nodules (Sensitivity 93%). Three nodules not identified: one nodule palpable (15 mm), second one not visible although palpable (5 mm), the third one (20 mm) neither visible nor palpable requiring conversion to an open procedure	VATS-US may have a role in revealing lesions occult at preoperative work-up
Messina et al. (81) - 2022, Italy. Retrospective study - *N* = 15 patients with small GGOs lesions. - Excluded patients: history of asthma, pulmonary fibrosis, chronic obstructive pulmonary disease or severe heart disease - CT detected 15 GGOs (6 pure and 9 mixed).	- MHz probe - VATS-US: Performed by an ultrasound-trained thoracic surgeon Investigated 15 lesions Identified 15 lesions, but 2 of them (in which the lung was not completely collapsed) after multiplanar CT reconstruction (Sensitivity 100% in collapsed lung). Mean size 14.1 ± 0.5 mm - Distance from pleural surface:4.8 ± 0.3 mm	- Small GGOs lesions: - Different types of echogenicity could also be the result of artefacts: In 13 patients with complete lung collapse, ultrasound produced clear images. In patients in whom the lung was not completely collapsed, the lesion could not be identified by ultrasound. Six pure lesions (40%) had hyperechoic patterns (due to residual air in the alveoli without stromal invasion) Nine mixed lesions (60%) had mixed echogenicity (hyperechoic mixed with hypoechoic patterns, due to the presence of air and a solid component) - Acoustic shadows were attributed to high internal echo images, whereas the acoustic enhancements were ascribed to low internal echo images. - The presence of a posterior echo was associated with the size of the GGO; larger GGOs exhibited a larger after-echo. - Hyperechoic pattern predominated over the hypoechoic component in the pre-invasive lesions and in the minimally invasive adenocarcinomas - The hypoechoic pattern predominated in the invasive adenocarcinoma - Ultrasound could not differentiate histologic subtypes of adenocarcinoma from ultrasound patterns
Piolanti et al. (7) - 2003, Italy. Prospective study - *N* = 35 patients, 40 nodules - No patients excluded - CT and PET detected 40 nodules [mean diameter 13.2 ± 5.9 mm (range 3–22), and mean distance from pleural surface was 16.10 ± 14.65 mm (range 0–54 mm)]	- 5–7.5 MHz linear probe with flexible tip - VATS-US: Performed by the surgeon under the guidance of the radiologist Investigated 40 nodules VATS-US identified 37 nodules (Sensitivity 92.5%). One lesion was not visualized due to its superficial and calcified nature, with a shadow cone indistinguishable from artifacts caused by missing sound transmission in the surrounding parenchyma. Another lesion (5 mm) was confused with a bronchial structure because it was hyperechoic. The third lesion was isoechoic from surrounding parenchyma	- Primitive malignant lesions (12 cases) appeared hypoechoic - Metastases showed a greater variability resulting hypoechogenic in most cases (50%); hypoechoic from surrounding parenchyma (22%); hyperechoic (22%), and fully calcified (6%) - The 12 benign lesions showed a greater variability in the sonographic pattern Complete pulmonary collapse occurred in 12 patients only - When lung collapse is not complete, the lesions appear as hypoechoic nodules with dorsal sound enhancement. - The thickness of the collapsed lung ranges from a few mm at the apex to 30 mm in the perihilar region, so a minimal amount of residual air does not hinder lesion localization.
Rocco et al. (82) - 2011, Italy. Prospective study - *N* = 2 patients with peripheral nodules - Excluded patients: previous history of extrathoracic cancer - CT detected 2 nodules	- 5–10 MHz articulated probe - VATS-US (uniportal) Performed by a radiologist with particular expertise in ultrasonography Investigated 2 nodules Identified 2 nodules (10 and 25 mm by histologic confirmation	Uniportal VATS-US can be successfully used to identify peripheral nodules.
Schauer et al. (63) - 2023, Germany. Prospective study - *N* = 30 patients - No excluded patients - CT detected 30 nodules	- 6–9 MHz probe - VATS-US: Performed by an experienced radiologist with over 10 years of expertise, working alongside the operating thoracic surgeons under sterile conditions. Investigated 30 nodules Identified 30 nodules (Sensitivity 100% by Io-CEUS)	- There are significant differences in vascularization as well as in contrast agent behavior depending on the tumor entity. - Io-CEUS may influence the surgeon's intraoperative decisions and the extent of lung resection. - Primary lung carcinomas (16) had jagged margins in B-mode and were predominantly inhomogeneous in echogenicity. Necrotic areas were present especially in larger tumors (>3 cm) and after neoadjuvant chemotherapy (*n* = 1). Power Doppler showed both peripheral and central macrovascularization. Io-CEUS showed an early peripheral and central wash in (mean time = 8.8 s ± 3) and a maximal distribution mean time 20 s, excluding the necrotic areas - Metastases (11). B-mode: Partly smooth and partly blurred border. Echogenicity was mostly hypoechogenic but sometimes echo-inhomogeneous. Power Doppler showed peripheral macrovascularization and in some of these patients it was even detected ubiquitously (no macrovasculariztion in 2 patients). Io-CEUS displayed marginal contrast enhancement in most cases - Benign nodule (3). Partly sharp but also partly blurred border and showed hypoechogenic to echo-inhomogeneous ultrasound pattern. Power Doppler demonstrated marginal macrovascularization in 2 cases and ubiquitous in 1 case. Io-CEUS showed early (4 s) and diffuse contrast enhancement with a subtomtal spread in 2 cases. In another case, late (29 s) and irregular enhancements were detected
Sperandeo et al. (48) - 2019, Italy. Prospective study - *N* = 14 patients. - No patient excluded - CT detected 14 pulmonary lesions	- 8–12 MHz linear probe with flexible tip - VATS-US: Investigated 14 pulmonary lesions Identified 14 pulmonary lesions (Sensitivity 100%)	- Correlation between echo-histologic type: - Cancer nodules (3 cases). Transthoracic ultrasound (TTU): mixed echostructure with defined margins. VATS-US: uniform hypoechogenic pattern nodule with jagged margins in 2 adenocarcinomas; and with defined margins in 1 patient with fibrosis (thick hyperechoic pleural line) and squamous carcinoma. - Insterstitial fibrosis (8 cases). TTU: irregular thickening of the hyperechoic pleural line (>3 mm) with an increase in the number of B-line artifacts. VATS-US: thick hyperechoic line - Hamartochondroma (1): hyperechoic nodule with defined margins. - Histocytosis X (2). TTU: mixed hypo-hyperechogenic structure with delineated margins.VATS-US: hypoechoic nodule and well defined margins - In general, and by VATS-US, cancer presents a uniform hypoechogenic pattern with jagged margins, and benign lesions present mixed hyperechoic structure of the margins.
Sortini et al. (38) - 2003, Italy. Prospective study. - *N* = 15 patients by VATS-US - Patient excluded: patients with nodules larger than 1.5 cm or < 2 cm deep - CT detected 14 pulmonary lesions	- 5.8 MHz linear probe - VATS-US: Investigated 15 pulmonary nodules Identified 15 pulmonary lesions (Sensitivity 100%) Size: 1.0 ± 0.14 cm (6 cases) and 1.4 ± 0.12 cm (9 cases) - Depth from pleural surface: 2.2 ± 0.20 cm (8 cases) and 3.1 ± 0.13 cm (7 cases)	- Nodules ≤1.5 cm or ≥ 2 cm deep - VATS-US seems superior to radioguided and finger palpation localization techniques for single pulmonary nodules.
Taurchini et al. (46) - 2021, Italy. Prospective study - *N* = 131 patients. 49 undergoing VATS-US with single nodules - Excluded patients: myocardial infarction or unstable angina; impossibility to tolerate single lung ventilation; uncooperative behavior - CT detected 131 nodules. The average diameter of the nodules was 12.50 ± 2.19 mm	- 8–12.5 MHz linear probe with a flexible tip - VATS-US: Investigated 49 nodules Identified 49 nodules (Sensitivity 100%) - The average diameter was 12.72 ± 2.27 mm (size was defined as the mean between the maximum and minimum diameter of the nodule)	- Ultrasonography provided a clear definition of the nodule margins, which generally corresponded to the true histological margins - Malignant nodules were hypoechoic in 96.67% of cases, with jagged margins (68.33%). Internal hyperechoic spots were found in 21.65% of cases. A regular rounded shape was observed in 53.33% of cases - Metastases were hypoechoic in 94.59%, with jagged margins (100.00%) - Benign nodules were hypoechoic in 94.12% of cases, with internal hyperechoic spots in 38.24%, and regular rounded shape in all cases, and no jagged margins in 91.18% of cases
Yamamoto et al. (64) - 2003, Japan. Prospective study - *N* = 26 patients - No excluded patients - CT detected	- 7.5 MHz flexible probe - VATS-US: Performed by two dedicated thoracic surgeons with over 5 years of experience. Investigated 26 nodules Identified 21 nodules (Sensitivity 81%). In 5 cases no visualized by US: One case retained air in the emphysematous lung, and in 4 cases the lesions were exceedingly deep - Nodule size 8–40 mm (mean 19,5 ± 8,5)	Peripheral pulmonary nodules - Primary lung cancer: isoechoic and uniform in 3 lesions and isoechoic and mixed in 9 lesions. Margin well defined and regular in 1 lesion, well defined and irregular in 10 lesions and ill-defined and irregular in 1 lesion. Intratumoral blood flow signal was high. The peak velocity was 26 ± 12.8 cm/s - Metastases: Hypoechoic and uniform in 2 tumors, hypoechoic and mixed pattern in 2 tumors and isoechoic and mixed patterns in the remaining 2 tumors. Margins well defined and regular in 3 lesions. irregular in 2 lesions and ill-defined in 1 lesion. The intratumoral blood flow signal was recognized in 4 of the 6 metastases. Intermediate grade of intratumoral blood flow. The peak velocity was 9.4 ± 1.7 cm/s - Benign: Uniform echogenicity and hypoechoic surrounded by normal lung echoes. Well-defined and regular margins. Tumoral blood flow signal was low or not recognized - Sonographic guidance during VATS aids in locating lesions and determining the extent of surgical resection - The Color Doppler method was useful for evaluating intratumoral blood flow, providing significant information for differentiating primary lung cancer, metastatic tumors, and various benign tumors

### Correlation of pulmonary nodule characteristics obtained by LU with preoperative CT and histological analysis data

The size of pulmonary nodules identified by intraoperative ultrasound is often correlated with the size observed on preoperative CT scans. For example, Hou et al. (40) found that the mean diameter of pulmonary nodules detected by LU was significantly correlated with the mean diameter obtained by CT (R = 0.860, *P* < 0.05) (ranging from 5 to 13.5 mm by LU and 8–20 mm by CT). However, other authors observed that the size range of ground-glass opacities (GGO) detected by LU was significantly smaller than that found by CT (23). Furthermore, the size and margins of pulmonary nodules identified by LU were correlated with data obtained from histological analysis (6, 23, 51, 54). According to some authors, this strong correlation may be partly due to the use of high-frequency ultrasound probes (55). Other authors have reported a weaker correlation between intraoperative ultrasound and histological analysis regarding the depth of the lung lesion (51). The pressure applied by the ultrasound probe on the lung surface of the sample, intended to eliminate residual peritumoral air and improve the visualization of the GGO, is likely responsible for this reduced correlation (55).

### Effectiveness of LU for localization of GGOs

Particular attention should be given to the location of GGOs. This term refers to the tomographic appearance of a focal opacity characterized by increased density in the lung parenchyma. Nodules detected by CT can be classified as solid or subsolid, with subsolid nodules further divided into partially solid nodules and GGOs. GGOs represent a hazy area of increased pulmonary attenuation while preserving bronchial and vascular markings. These lesions can be either mixed or pure, depending on the presence of a solid component within (56). GGOs encompass benign conditions, such as infections, as well as malignancies, including lung cancer (adenocarcinoma). Opacities smaller than 1 cm are typically indicative of atypical adenomatoid hyperplasia or adenocarcinoma *in situ*, whereas lesions exceeding this size increase the likelihood of invasive adenocarcinoma, with the risk rising from 10% to 50%. Their malignant and invasive potential is further elevated when a solid component is present within the lesion (57).

Many GGOs, unlike solid tumors, are not easily detected during VATS surgical procedures (7, 22, 55, 58). Aside from the fact that they are generally not palpable, the density of these lesions is similar to that of the adjacent normal parenchyma, making their sonographic identification challenging even for experienced surgeons, especially when dealing with pure GGOs (those with no solid component) or deeply located GGOs (51). Furthermore, their identification in the specimen can pose technical challenges for pathologists and may require sectioning and microscopic examination of the entire specimen. In such cases, marking the lesion with an ultrasound-guided needle can facilitate rapid localization by the pathologist, reducing the number of sections needed and shortening operative time. This approach is less cumbersome than CT-guided needle identification of lung nodules in reaerated lung specimens. When using this technique, in addition to a radiology room, moderate mechanical aeration is required to enable the detection of GGO with CT. This, however, is not feasible when the sample is compromised by air leaks (59).

In some GGOs, a posterior echo may be observed, which could assist in their identification and might be related to the size of the GGO; however, further studies are needed to confirm this finding (23, 60). Achieving a complete lung collapse, as well as using electrocautery to mark the visceral pleura and better define the margins of the lesions, can be helpful when resecting these tumors (23, 51). Kondo et al. (23) considered resections with margins 1.5 times the diameter of the lung nodule, or at least 2 cm, as safe. Other authors have proposed preoperative marking of tumors for pulmonary nodules ≤10 mm in size, located more than 5 mm from the pleural surface, as they observed intraoperative detection failures of up to 63% (61). Jian Hu et al. (62) also advocated for marking with methylene blue, 2 hours before thoracoscopic wedge resection, those nodules that, on CT, showed a distance greater than 1.5 cm from the pleural surface and were difficult to identify due to their location. Despite the challenges in identifying these tumors, some authors, such as Fiorelli et al. (51), were able to identify 100% of GGOs in an ex vivo analysis, with an average size of 13.9 ± 0.5 mm, and in 11 of 17 (65%) cases, the lesions were smaller than 15 mm, with a mean depth of 4.4 ± 0.3 mm. In all cases, pathologists were able to conclusively identify these lesions with the initial analysis.

## Effectiveness of ultrasound in determining the severity of injuries

### Based on echogenicity patterns

Some authors have distinguished between primary lesions, metastases, and benign lesions based on various ultrasound patterns. Regarding primary tumors, Khereba et al. (39) reported no correlation between the ultrasound patterns and the malignancy or benignity of the lesions. Conversely, other studies (39, 52) identified a heterogeneous pattern with punctile hyperechoic areas and ill-defined or jagged margins in primary tumors. Schauer et al. (63) noted well-defined necrotic areas in larger tumors (>3 cm), potentially contributing to the observed punctile hyperechoic pattern. Similarly, Piolanti (7) and Sperandeo et al. (48) found a predominance of hypoechoic lesions with jagged margins, while Yamamoto et al. (64) described these as predominantly isoechoic with irregular, well-defined margins.

In the case of metastatic lesions, some authors could not distinguish between primary or metastatic origins based on ultrasound patterns (37, 48). Piolanti et al. (7) found that 50% of metastatic lesions were hypoechoic, while the remaining showed a more variable pattern, with some appearing hyperechoic. Schauer et al. (63) similarly reported that most metastases were hypoechoic, occasionally non-homogeneous, with both smooth and blurred edges. Yamamoto et al. (64) observed a predominance of hypoechoic patterns in metastases, with margins ranging from well-defined to irregular. Benign lesions displayed significant variability in ultrasound patterns, appearing as both hyperechoic and hypoechoic, typically with well-defined margins (37, 48, 64).

Regarding GGOs, Hou et al. (40) found no correlation between ultrasound patterns and the malignancy or benignity of the lesions. However, Fiorelli et al. (51) concluded that pure and mixed GGOs with predominantly hyperechoic patterns were associated with preinvasive or minimally invasive lesions, while those with hypoechoic patterns indicated invasive adenocarcinoma. Kondo et al. (23) found that the echogenicity of GGOs, whether hyperechoic or hypoechoic, was influenced by the degree of lung aeration during lung collapse. Complete lung collapse typically resulted in a hyperechoic pattern, whereas incomplete collapse produced a mixed or speckled hyperechoic pattern due to residual air.

### Based on the Doppler echocardiographic record

Several researchers have identified a correlation between the type of lesion -whether malignant or benign- and its vascularization pattern as observed through Color Doppler imaging. In cases of primary lung carcinomas, Schauer et al. (63) demonstrated the presence of both peripheral and central macrovascularization using Color-Coded Doppler Sonography (CCDS) or Power Doppler techniques. Similarly, Yamamoto et al. (64) reported a significant intratumoral blood flow signal in all primary lesions, with a peak velocity averaging 26 ± 12.8 cm/s. Notably, this elevated blood flow was particularly pronounced in eight out of twelve examined lesions.

In the context of metastasis, Schauer et al. (63) observed a predominance of peripheral macrovascularization in these lesions, with some cases exhibiting either ubiquitous or even undetectable vascularization. Yamamoto et al. (64) found a medium-grade intratumoral blood flow signal in four of the six metastatic lesions studied, with a peak velocity of 9.4 ± 1.7 cm/s, marking a statistically significant contrast to the higher velocities seen in primary tumors.

For benign lesions, the findings were distinct. Schauer et al. (63) documented marginal macrovascularization in two cases and ubiquitous macrovascularization in one. In contrast, Yamamoto et al. (64) found no evidence of tumor flow signals in benign lesions, suggesting a markedly different vascular profile when compared to malignant tumors. These observations underscore the potential of Color Doppler imaging as a diagnostic tool in distinguishing between different types of pulmonary lesions based on their vascular characteristics.

### Based on the results obtained by Io-CEUS

Pulmonary nodules were further characterized by the pattern of contrast distribution, specifically regarding peripheral and/or central enhancement, using Io-CEUS. Schauer et al. (63) explored the application of Io-CEUS in identifying pulmonary nodules that were challenging to visualize and characterize before resection. In their study, bolus injections of 2.4–5 ml sulfur hexafluoride microbubbles (SonoVue®, Bracco, Milan, Italy) were administered, followed by 10–20 ml of NaCl, delivered through a central venous catheter by the anesthesiologist. The behavior of the contrast agent varied significantly with the tumor type. In primary lung carcinomas, early peripheral and central washout was observed, with a mean time of 8.8 s ± 3, and a mean maximum distribution time of 20 s, excluding necrotic areas when present. For metastatic lesions, Schauer et al. (63) noted marginal contrast enhancement in most cases; the timing of contrast enhancement varied, occurring early in some cases (t = 6 s) and late in others (t = 16 s). For benign nodules, Io-CEUS demonstrated early (as early as 4 s) and diffuse contrast enhancement in two cases, while in another case, it revealed late (29 s) and irregular enhancement. These findings highlight the potential of Io-CEUS in differentiating pulmonary nodule types based on their contrast enhancement patterns, offering valuable real-time intraoperative insights.

## Determining tumor resectability using ultrasound

The use of intrathoracic ultrasound to assess tumor resectability has been explored in limited studies. Santambrogio et al. (55) investigated the cardiovascular and metastatic invasion of malignant lung lesions. Upon identifying the lung nodule, they evaluated its proximity to the vessels and heart by methodically moving the ultrasound probe from the upper to the lower mediastinum and towards the hilum, considering lymph nodes measuring 15 mm or more as indicative of metastasis. Lesser et al. (65) also assessed the resectability of 155 nodules, all classified as non-small cell lung cancer (NSCLC, T4 N0-1) and meeting criteria for invasiveness. In all patients, CT scans revealed close contact between the tumor and mediastinal structures (T4), such as the pulmonary artery, left or right atrium, superior vena cava (SVC), and thoracic aorta, raising concerns about operability. The ultrasound probe was strategically placed over hilar and mediastinal structures to evaluate operability criteria, which included: less than one-third circumference infiltration of the SVC without right atrium invasion, no aortic wall infiltration, at least 1 cm of tumor-free intrapericardial pulmonary artery, and less than 1 cm invasion of the left atrium near the origin of the pulmonary veins. The pulmonary lesion locations were accurately established in all patients. Resectability staging using VAT-US demonstrated a sensitivity of 94.1% and a specificity of 98.1%. However, false negative and false positive rates for the pulmonary artery and left atrium were notably high (16.1% and 14.8%, respectively). The study suggested that positioning the probe over the pulmonary hilum in various planes and intrapericardially could enhance the assessment of tumor infiltration in the left atrium and heart. Employing high-frequency ultrasound probes was also recommended for improved accuracy. The authors concluded that utilizing ultrasound to determine operability could be beneficial for patients deemed inoperable, potentially reducing the need for diagnostic thoracotomies.

## Pulmonary collapse for the identification of pulmonary nodules using lung ultrasound

During lung surgery, the target lung is typically deflated, often through the use of a lung ventilation device that enables single-lung ventilation. This process reduces the air content in the bronchoalveolar spaces of the excluded lung, facilitating deeper exploration with an ultrasound probe. In fully deflated lungs, pulmonary arterioles and venules appear as homogeneous hypoechoic areas, while bronchioles are visualized as hyperechoic spots, enhancing the identification of lung nodules.

Khereba and Kondo et al. (23, 39) proposed that the varying echogenicities observed during intraoperative LU recordings could be attributed to artifacts rather than the size, histological type of the lesions, or the presence of solid components. In a fully deflated lung, GGOs typically present a hyperechoic pattern. However, if the lung is not entirely deflated, as might occur in cases of chronic obstructive pulmonary disease, the internal lung structures are likely to exhibit a “mottled hyperechoic pattern” due to residual air echo artifacts. Additionally, dorsal sound enhancement may be noted around lung lesions, resulting from the greater transmission of the ultrasound beam through the nodules compared to the surrounding parenchyma, where ultrasound reflection is more pronounced due to residual air (23). Nevertheless, a minimal presence of residual air should not significantly hinder lesion identification (55), as the thickness of the collapsed lung ranges from a few millimeters at the apex to 30 mm in the perihilar region, with residual air primarily limiting studies beyond a depth of approximately 20 mm.

Anesthesiologists can enhance the ultrasound image by minimizing the amount of air within the lung parenchyma. This can be accomplished through the proper placement of the double-lumen tube (DLT). Incorrect placement, which occurs in up to 14% of cases, may result in intraoperative desaturation and inadequate lung protection against hemorrhage, pus, and secretions (66). The replacement of these tubes should be carried out with caution due to the potential complications, such as tracheal injury, which may include irritation, laryngitis, or airway rupture. The membranous part of the trachea (52%) and the left main bronchial trunk (37%) are the most common sites of rupture (67). These complications are more frequent in patients who have undergone radiotherapy or corticosteroid treatment, which weakens the tracheobronchial wall and contributes to several risk factors (53, 68). These factors include difficulties in intubation, multiple attempts at repositioning the DLT, use of oversized tubes, or overinflation of the bronchial cuff, leading to mucosal ischemia. Therefore, a proper lung collapse should be confirmed using a fiberoptic bronchoscope, and the correct choice of DLT size is essential (66). Preoperative CT scans can aid in selecting the appropriate DLT size by assessing the diameters of the main left bronchus and cricoid cartilage (69). Transthoracic ultrasound is also a valuable tool in DLT sizing, as it allows for the measurement of the trachea's external diameter or the cricoid (70, 71) and confirms the correct positioning of the left DLT (72). Furthermore, transthoracic ultrasound can be used to assess lung aeration after DLT placement, facilitating ventilation optimization. Loss of lung aeration can be visualized through B-lines or vertical hyperechoic lines emanating from the pleural line, which move with respiration. The separation of these lines indicates moderate loss of aeration, while their merging suggests severe loss due to alveolar filling. The absence of diaphragmatic and pleural movement also signals lung collapse (73–79).

However, despite the correct selection of the DLT, complete lung isolation cannot always be assured, particularly in patients with severe chronic obstructive pulmonary disease (COPD) (45), which can introduce errors in ultrasound evaluation (21–23). Some authors, while emphasizing the importance of deflating the lung, were unable to achieve full lung deflation in 30% of patients, even after applying gentle pressure to the lung surface with an instrumental probe (23). For this reason, patients with asthma and COPD were excluded from some ultrasound studies. Additionally, the time required to achieve complete lung exclusion is not negligible, with an average time of 40 minutes documented from the selective exclusion of the affected lung (45). Furthermore, it must be acknowledged that in some cases, maintaining proper single-lung ventilation to ensure tissue oxygenation and patient stability may be difficult or even impossible.

## Discussion

Data from the scientific literature show the usefulness of VATS-US in the identification of both very small peripheral pulmonary nodules [<2 mm (37)] and deep nodules (38, 45, 80), allowing safe and small resection margins and decreasing conversion to thoracotomy (39). In addition, performing LU does not entail complications such as those described in other nodular identification techniques (insertion of metals with CT-guided wire hook, injection of methylene blue or isotopes, intraoperative endoscopic tracheal magnetic navigation, among others). No patient presented arrhythmias, changes in blood pressure, bleeding or other complications during the examination performed with intrathoracic ultrasound, so it is considered a safe technique, in addition to being able to be performed in real time and at low cost. In addition, Colour Doppler and Io-CEUS have helped to differentiate lung lesions according to their benignity/malignancy, although further studies are needed to define and confirm the different vascular patterns and contrast distribution (63, 64). With the data currently available, primary lesions present both central and peripheral vascularization; in metastases, vascularization seems to have a peripheral predominance, and in benign lesions both marginal macrovascularization and avascularization have been described. This vascular distribution was correlated with the different patterns of Io-CEUS contrast distribution in the lesions. Thus, primary lung carcinomas showed early peripheral and central washout, metastases showed marginal contrast enhancement and benign lesions showed more diffuse enhancement. Confirmation of these results could allow what some authors have called “living histology” in thoracic surgery, potentially influencing decision-making on the extent of lung resection in the future (63). Nevertheless, given that the pulmonary vasculature is influenced by lung collapse, it should be considered that the use of contrast-enhanced ultrasound for the characterization of pulmonary lesions remains controversial. Further research is required to determine its degree of utility, as the existing literature is limited. However, despite the impact of lung collapse on normal pulmonary perfusion, the residual perfusion of the bronchial arteries in primary or metastatic tumors may be utilized to enhance intraoperative localization. Its implementation as a complementary tool in VATS could optimize surgical precision and reduce the morbidity associated with unnecessary resections.

Finally, VATS-US has also been shown to be useful in staging the resectability of T4 non-small cell lung cancer (NSCLC). Resectability depends on the degree of tumor involvement of mediastinal structures, and CT does not always allow distinction between tumor bordering the mediastinum and tumor invading mediastinal structures. By allowing mediastinal dissection, albeit to a limited extent, VATS-US has been shown to be useful in determining whether tumor has infiltrated the descending aorta, superior vena cava, or right atrium. It has also allowed determination of the exact margin of tumor infiltration in the left atrium (65). However, it should be considered that, currently, PET-CT and MRI remain the gold standard for evaluating mediastinal invasion. Intraoperative ultrasound can play a complementary role in the intraoperative assessment, for example, in helping to define the degree of invasion or identify abnormal vasculature, but it should not replace these advanced techniques.

The application of VATS-US has certain limitations. The presence of COPD or heart disease can make it challenging to identify pulmonary nodules via ultrasound. In such cases, as well as in situations involving GGOs and non-peripheral lung lesions smaller than 10 mm in diameter (located more than 5 mm from the pleural surface), preoperative marking may be considered (23). Furthermore, there are other factors that can lead to incorrect ultrasound interpretation, such as recent myocardial infarction or unstable angina, pulmonary edema, pleural retraction, pulmonary fibrosis, among others. Thus, several authors choose to exclude these patients from their studies (23, 39, 52). Moreover, the interpretation of images is subjective and requires specialized training, education, and experience—factors that significantly limit the use of this technique. Although some authors suggest that VATS-US is a straightforward technique with a rapid learning curve (39), most published studies indicate that VATS-US was performed by thoracic surgeons, sometimes guided by radiologists, or by radiologists with over 10 years of experience (63) (Table 1).

Additionally, drawing conclusions about the benignity or malignancy of lesions based solely on the echogenicity pattern is challenging. Typically, primary lesions are predominantly heterogeneous, with mixed echogenicity (hypoechoic and hyperechoic areas) and irregular margins. In metastases, a hypoechoic pattern with irregular margins is predominant, although to a lesser extent than in primary tumors. In benign lesions, there is significant variability in echogenicity, with well-defined margins being more common. However, these criteria alone do not confirm the benignity or malignancy of the lesions, and histological confirmation is necessary.

Moreover, scientific literature highlights a lack of homogeneous ultrasound criteria for defining lung lesions, complicating their characterization. It is essential to distinguish between concepts such as hyperechogenicity and heterogeneity, or hypoechogenicity and homogeneity, as well as to clearly define the margins of lesions, specifying whether they are irregular or well-defined. When describing ultrasound lesions, it is also necessary to mention the degree of lung collapse achieved, as residual air can create artifacts (81) and errors in the ultrasound interpretation of lesions. This residual air depends not only on the correct placement and inflation of the DLT but also on the intrinsic characteristics of patients, particularly whether they suffer from respiratory or cardiac conditions.

In summary, the identification of pulmonary nodules can be enhanced by implementing specific measures. Firstly, the preoperative identification of pulmonary lesions via CT is crucial. Performing VATS-US does not eliminate the need for intraoperative visual inspection and palpation of pulmonary lesions identified by CT (22, 23). The selection of the most appropriate probe for the lesion to be resected should be based on its depth. High-frequency ultrasound probes (>5 MHz) are recommended for visualizing more superficial or smaller tumors (≤2 mm), while low-frequency probes (3.5 MHz) are preferable for deeper lesions (>4 mm) (46, 81). Additionally, the use of ultrasound probes with flexible tips facilitates multiplanar exploration of the area of interest (22, 82), making it necessary to insert the ultrasound probe between the fissures to visualize the lung parenchyma from all angles. To optimize the imaging, complete lung deflation is essential. Inducing lung collapse prior to lesion excision, when the patient is positioned for surgery, can promote greater lung deflation. Moreover, careful air suction through the DLT and the application of pressure on the lung by the ultrasound probe help release lung air and facilitate lung collapse (6, 23). It is important to note that the pressure exerted by the ultrasound probe can influence the precise measurement of the distance between the lesion and the pleural surface. Once the lung has collapsed, filling the pleural space with saline (500 ml) may enhance ultrasound definition (23). Finally, the success of the VATS-US technique relies heavily on the practitioner's learning and experience (63).

Further research is needed to enhance the VATS-US technique, thereby enabling a more reliable characterization of lung lesions in terms of their benignity or malignancy. In this context, artificial intelligence could play a pivotal role by integrating various algorithms that take into account not only the tumor's features—such as echogenicity, margins, vascular patterns, and contrast distribution—but also the patient's intrinsic characteristics that may influence the degree of lung collapse. Additionally, ethnic and regional variations, which may imply differences in lung lesions, should be considered in the development of these algorithms.
